# Supplementary material for: Multi‐dataset identification of innovative feature genes and molecular mechanisms in keratoconus
Source: J Cell Mol Med. 2024 Sep 19;28(18):e70079. doi: 10.1111/jcmm.70079 (PMC11412914; doi:10.1111/jcmm.70079)
Supplement: Supplementary file 2 — Material S2. [file JCMM-28-e70079-s002.zip › 7.GSEA analysis/5.RNF217-AS1/KEGG_OOCYTE_MEIOSIS.html]

Details for gene set KEGG\_OOCYTE\_MEIOSIS[GSEA]

|  || Dataset | RNF217-AS1.group.cls#h\_versus\_l |
| Phenotype | group.cls#h\_versus\_l |
| Upregulated in class | h |
| GeneSet | KEGG\_OOCYTE\_MEIOSIS |
| Enrichment Score (ES) | 0.14424929 |
| Normalized Enrichment Score (NES) | 0.73616505 |
| Nominal p-value | 0.80923694 |
| FDR q-value | 1.0 |
| FWER p-Value | 1.0 |
Table: GSEA Results Summary

  

Fig 1: Enrichment plot: KEGG\_OOCYTE\_MEIOSIS      
 Profile of the Running ES Score & Positions of GeneSet Members on the Rank Ordered List

  

| SYMBOL | TITLE | RANK IN GENE LIST | RANK METRIC SCORE | RUNNING ES | CORE ENRICHMENT || 1 | CALML6 | na | 1514 | 0.391 | -0.0035 | Yes |
| 2 | PPP3R2 | na | 1532 | 0.389 | 0.0360 | Yes |
| 3 | FBXO43 | na | 2815 | 0.300 | 0.0297 | Yes |
| 4 | ADCY2 | na | 2970 | 0.293 | 0.0554 | Yes |
| 5 | CAMK2A | na | 3603 | 0.267 | 0.0646 | Yes |
| 6 | ADCY4 | na | 4384 | 0.238 | 0.0665 | Yes |
| 7 | CPEB1 | na | 4406 | 0.237 | 0.0903 | Yes |
| 8 | ADCY5 | na | 4777 | 0.225 | 0.1027 | Yes |
| 9 | CAMK2B | na | 5000 | 0.217 | 0.1186 | Yes |
| 10 | CHP2 | na | 5296 | 0.206 | 0.1312 | Yes |
| 11 | ADCY7 | na | 5579 | 0.197 | 0.1433 | Yes |
| 12 | IGF1R | na | 6917 | 0.155 | 0.1206 | Yes |
| 13 | RPS6KA2 | na | 7112 | 0.150 | 0.1304 | Yes |
| 14 | CCNE1 | na | 7161 | 0.148 | 0.1442 | Yes |
| 15 | MAPK12 | na | 9095 | 0.103 | 0.0990 | No |
| 16 | PPP2R1B | na | 9136 | 0.102 | 0.1084 | No |
| 17 | YWHAE | na | 10174 | 0.083 | 0.0870 | No |
| 18 | ESPL1 | na | 10529 | 0.077 | 0.0847 | No |
| 19 | ADCY1 | na | 10624 | 0.076 | 0.0898 | No |
| 20 | PPP2R5B | na | 11671 | 0.060 | 0.0658 | No |
| 21 | SMC1B | na | 12302 | 0.051 | 0.0528 | No |
| 22 | PPP2R5C | na | 12306 | 0.051 | 0.0580 | No |
| 23 | ITPR3 | na | 12500 | 0.049 | 0.0575 | No |
| 24 | ANAPC1 | na | 12601 | 0.047 | 0.0594 | No |
| 25 | PTTG2 | na | 12847 | 0.044 | 0.0569 | No |
| 26 | STAG3 | na | 13138 | 0.040 | 0.0527 | No |
| 27 | YWHAZ | na | 13310 | 0.038 | 0.0517 | No |
| 28 | IGF1 | na | 13325 | 0.038 | 0.0552 | No |
| 29 | MAPK1 | na | 13362 | 0.038 | 0.0581 | No |
| 30 | SMC1A | na | 13405 | 0.037 | 0.0607 | No |
| 31 | YWHAQ | na | 13522 | 0.036 | 0.0610 | No |
| 32 | PTTG1 | na | 13701 | 0.034 | 0.0593 | No |
| 33 | CALM2 | na | 13884 | 0.032 | 0.0574 | No |
| 34 | PPP3CB | na | 13986 | 0.031 | 0.0576 | No |
| 35 | CCNE2 | na | 14005 | 0.031 | 0.0602 | No |
| 36 | MAPK3 | na | 14127 | 0.029 | 0.0598 | No |
| 37 | PGR | na | 14182 | 0.029 | 0.0611 | No |
| 38 | CDK2 | na | 14696 | 0.022 | 0.0486 | No |
| 39 | RPS6KA3 | na | 14828 | 0.021 | 0.0470 | No |
| 40 | PPP2R5A | na | 14850 | 0.021 | 0.0485 | No |
| 41 | ANAPC5 | na | 15025 | 0.019 | 0.0454 | No |
| 42 | MOS | na | 15405 | 0.015 | 0.0360 | No |
| 43 | SLK | na | 15431 | 0.015 | 0.0368 | No |
| 44 | CALML5 | na | 15459 | 0.014 | 0.0375 | No |
| 45 | PPP2CB | na | 15463 | 0.014 | 0.0389 | No |
| 46 | CAMK2G | na | 16021 | 0.008 | 0.0236 | No |
| 47 | CDC23 | na | 16140 | 0.007 | 0.0210 | No |
| 48 | RBX1 | na | 16317 | 0.006 | 0.0165 | No |
| 49 | FBXW11 | na | 16324 | 0.006 | 0.0169 | No |
| 50 | YWHAG | na | 16384 | 0.005 | 0.0157 | No |
| 51 | PPP2R5D | na | 16548 | 0.003 | 0.0113 | No |
| 52 | SKP1 | na | 16603 | 0.003 | 0.0100 | No |
| 53 | ANAPC7 | na | 16717 | 0.001 | 0.0069 | No |
| 54 | PLCZ1 | na | 16755 | 0.001 | 0.0060 | No |
| 55 | ADCY9 | na | 17206 | -0.004 | -0.0067 | No |
| 56 | PPP2R5E | na | 17233 | -0.004 | -0.0070 | No |
| 57 | CALML3 | na | 17310 | -0.005 | -0.0087 | No |
| 58 | PPP2R1A | na | 17564 | -0.007 | -0.0153 | No |
| 59 | PPP1CB | na | 18110 | -0.012 | -0.0299 | No |
| 60 | ANAPC11 | na | 18123 | -0.012 | -0.0290 | No |
| 61 | CDC20 | na | 18128 | -0.012 | -0.0279 | No |
| 62 | CALM3 | na | 18318 | -0.014 | -0.0320 | No |
| 63 | CDC27 | na | 18332 | -0.014 | -0.0309 | No |
| 64 | YWHAB | na | 18544 | -0.016 | -0.0354 | No |
| 65 | SPDYC | na | 18610 | -0.016 | -0.0356 | No |
| 66 | PKMYT1 | na | 18680 | -0.017 | -0.0358 | No |
| 67 | ANAPC13 | na | 18874 | -0.019 | -0.0394 | No |
| 68 | RPS6KA1 | na | 18901 | -0.019 | -0.0382 | No |
| 69 | ADCY6 | na | 19355 | -0.024 | -0.0488 | No |
| 70 | BTRC | na | 19413 | -0.024 | -0.0480 | No |
| 71 | PPP2CA | na | 19715 | -0.027 | -0.0539 | No |
| 72 | CAMK2D | na | 20347 | -0.033 | -0.0687 | No |
| 73 | CDC16 | na | 20405 | -0.034 | -0.0668 | No |
| 74 | INS | na | 20462 | -0.035 | -0.0649 | No |
| 75 | ANAPC4 | na | 20735 | -0.037 | -0.0689 | No |
| 76 | CDK1 | na | 20745 | -0.037 | -0.0654 | No |
| 77 | PPP3R1 | na | 20845 | -0.038 | -0.0643 | No |
| 78 | CHP1 | na | 21142 | -0.042 | -0.0686 | No |
| 79 | PPP1CC | na | 21177 | -0.042 | -0.0652 | No |
| 80 | ANAPC10 | na | 21518 | -0.045 | -0.0704 | No |
| 81 | ADCY8 | na | 21903 | -0.050 | -0.0764 | No |
| 82 | CUL1 | na | 21972 | -0.050 | -0.0732 | No |
| 83 | ITPR2 | na | 21984 | -0.051 | -0.0683 | No |
| 84 | REC8 | na | 22224 | -0.053 | -0.0697 | No |
| 85 | PPP1CA | na | 22549 | -0.057 | -0.0733 | No |
| 86 | ADCY3 | na | 22794 | -0.059 | -0.0742 | No |
| 87 | PRKACA | na | 23066 | -0.063 | -0.0756 | No |
| 88 | MAP2K1 | na | 23215 | -0.064 | -0.0733 | No |
| 89 | SMC3 | na | 23865 | -0.073 | -0.0845 | No |
| 90 | MAD2L1 | na | 23884 | -0.074 | -0.0775 | No |
| 91 | PPP3CA | na | 24064 | -0.076 | -0.0748 | No |
| 92 | MAD2L2 | na | 24443 | -0.082 | -0.0774 | No |
| 93 | CDC26 | na | 24588 | -0.084 | -0.0729 | No |
| 94 | CCNB1 | na | 24923 | -0.089 | -0.0734 | No |
| 95 | YWHAH | na | 25403 | -0.097 | -0.0773 | No |
| 96 | ITPR1 | na | 25459 | -0.098 | -0.0688 | No |
| 97 | CCNB2 | na | 26396 | -0.114 | -0.0841 | No |
| 98 | PRKACB | na | 26457 | -0.116 | -0.0739 | No |
| 99 | CALM1 | na | 26557 | -0.117 | -0.0647 | No |
| 100 | SPDYA | na | 26678 | -0.119 | -0.0559 | No |
| 101 | PPP3CC | na | 26944 | -0.124 | -0.0508 | No |
| 102 | RPS6KA6 | na | 27330 | -0.133 | -0.0483 | No |
| 103 | ANAPC2 | na | 27696 | -0.141 | -0.0444 | No |
| 104 | BUB1 | na | 27755 | -0.143 | -0.0314 | No |
| 105 | AURKA | na | 29556 | -0.193 | -0.0636 | No |
| 106 | FBXO5 | na | 29726 | -0.198 | -0.0482 | No |
| 107 | PRKX | na | 31362 | -0.257 | -0.0690 | No |
| 108 | AR | na | 32445 | -0.320 | -0.0674 | No |
| 109 | PLK1 | na | 32706 | -0.338 | -0.0401 | No |
| 110 | PRKACG | na | 33174 | -0.374 | -0.0152 | No |
| 111 | CDC25C | na | 34368 | -0.585 | 0.0104 | No |
Table: GSEA details [plain text format]

  

Fig 2: KEGG\_OOCYTE\_MEIOSIS      
 Blue-Pink O' Gram in the Space of the Analyzed GeneSet

  

Fig 3: KEGG\_OOCYTE\_MEIOSIS: Random ES distribution      
 Gene set null distribution of ES for **KEGG\_OOCYTE\_MEIOSIS**
